# Geographic variation in floral traits and the capacity of autonomous selfing across allopatric and sympatric populations of two closely related *Centaurium* species

**DOI:** 10.1038/srep46410

**Published:** 2017-04-21

**Authors:** Dorien Schouppe, Rein Brys, Mario Vallejo-Marin, Hans Jacquemyn

**Affiliations:** 1 KU Leuven, Department of Biology, Plant Conservation and Population Biology, B-3001 Leuven, Belgium; 2 Research Institute for Nature and Forest, Kliniekstraat 25, BE-1070 Brussels, Belgium; 3 Biological and Environmental Sciences. University of Stirling. Stirling, FK9 4LA, Scotland, United Kingdom

## Abstract

Floral traits and the relative contribution of autonomous selfing to total seed set varies geographically and is often driven by the availability and abundance of suitable pollinators and/or the presence of co-flowering relatives. In the latter case, competition for pollinator services and costs of hybridization can select for floral traits that reduce interspecific gene flow and contribute to prezygotic isolation, potentially leading to geographic variation in floral divergence between allopatric and sympatric populations. In this study, we investigated variation in floral traits and its implications on the capacity of autonomous selfing in both allopatric and sympatric populations of two closely related *Centaurium* species(Gentianaceae) across two distinct geographic regions(UK and mainland Europe). Although the magnitude and direction of floral differentiation varied between regions, sympatric populations were always significantly more divergent in floral traits and the capacity to self autonomously than allopatric populations. These results indicate that mating systems can vary substantially within a species and that the joint occurrence of plant species can have a major impact on floral morphology and capacity of autonomous selfing, most likely as a way to reduce the probability of interspecific interference.

Plant species display large variation in floral morphology and mating systems along broad geographic regions^1^,^2^,^3^,^4^. In most cases, changes in mating system do not represent changes in a single trait, but involve changes in a suite of floral traits^4^,^5^. For example, when plants are subjected to severe pollen limitation, autonomous self-fertilization is expected to evolve as a strategy to provide reproductive assurance^6^,^7^. Under such conditions, floral traits that promote autonomous selfing, such as reductions in the level of herkogamy or dichogamy, are selected^6^,^8^,^9^,^10^,^11^,^12^. At the same time, selection for reduced investment in pollinator attraction, such as reductions in flower size, corolla diameter, number of flowers, floral longevity and nectar and/or pollen production, often co-occur^7^,^11^,^13^,^14^,^15^. Some of these changes may, in turn, allow more efficient selfing and at the same time incur little loss of resources that are otherwise used to attract pollinators.

In cases where closely related plant species co-occur, flower at the same time and share the same pollinators, differentiation in floral traits and mating system can result from selection pressures that prevent interspecific gene flow, especially when heterospecific pollen transfer and hybrid fertilization are costly^16^,^17^,^18^,^19^,^20^,^21^. Under such conditions, the evolution towards autonomous selfing is also one of the mating system transitions that may guarantee reproductive isolation. For instance, in the recent evolution of *Senecio eboracensis* from *S. vulgaris*, predominant self-fertilization in *S. eboracensis* contributed to strong reproductive isolation and ecological differentiation^22^. In *Mimulus nasutus* almost exclusive autogamy offered nearly complete isolation from its close relative *M. guttatus* when they were growing in sympatry^23^. Similarly, the combined reduction in herkogamy and dichogamy, resulting in a higher capacity for autonomous selfing, offered effective mechanical protection against heterospecific mating and played an important contribution to total reproductive isolation among three closely related *Centaurium* species^24^.

Apart from the autonomous selfing mechanism itself^25^,^26^, the reduced attractiveness of some selfing species may further lower the risk of being visited and pollinated with heterospecific pollen^27^. An associated reduction in pollen production may, on the other hand, reduce a species’ contribution to the potential total sympatric pollen pool, which can in turn increase the risk of being pollinated with heterospecific pollen when visited by a pollinator foraging on both plant species^28^. Although compelling evidence is still largely lacking, there are a few studies that have demonstrated that pollinator-mediated interspecific interference and competition have contributed to differentiation in floral traits and mating system^19^,^25^,^26^,^29^.

Due to the interplay of large-scale geographic variation in floral traits and small-scale variation resulting from species interactions in secondary contact zones, it can be predicted that a complex mosaic of floral traits and mating systems arises across large geographic areas. To test this prediction, we studied variation in floral traits and the capacity of autonomous selfing in both sympatric and allopatric populations of *Centaurium erythraea* and *C. littorale*, and investigated whether the same patterns hold across two distinct geographic regions. Both species display the same flower morphology, frequently co-occur in both regions and are known to hybridize under sympatric conditions in the field^30^,^31^,^32^,^33^,^34^. However, previous work has also shown that hybridisation is costly due to severe post-zygotic fitness reductions in terms of seed set and offspring performance^33^. Recent analyses have further shown that floral and mating system characteristics in *Centaurium* can differ significantly between contrasting pollinator environments and that floral traits may rapidly evolve to ensure seed production in pollinator-poor environments^11^. Comparisons of floral traits in sympatric populations of the two species in Belgium has indicated that *C. erythraea* is characterized by high levels of herkogamy and is predominantly outcrossing, whereas *C. littorale* shows little spatial separation between anthers and stigma and is largely selfing^35^. Earlier work by Ubsdell^36^ on the same species, on the other hand, showed exactly the opposite pattern for allopatric populations in the UK. He documented the highest levels of autonomous selfing in *C. erythraea* and a much lower capacity for autonomous selfing in *C. littorale*, which he attributed to a lack of herkogamy in *C. erythraea* flowers and stronger spatial stigma-anther separation in flowers of *C. littorale*.

Since heterospecific pollen transfer between *C. erythraea* and *C. littorale* and hybrid fertilization have been shown to be costly, in terms of both seed production and progeny fitness^33^, we hypothesized that in order to minimize the risk of hybridization, sympatric populations will tend to show stronger differentiation in floral morphology and selfing capacity than allopatric populations. To test this hypothesis, we compared floral traits and the capacity of autonomous selfing between allopatric and sympatric populations of both species. To investigate in more detail the extent to which differences in floral and mating system traits between allopatric and sympatric populations were affected by geographical location, we quantified geographic variation in floral traits and the contribution of autonomous selfing to total seed set in two contrasting regions, namely mainland Europe and the UK.

## Material and Methods

### Study species

**Figure 1:**
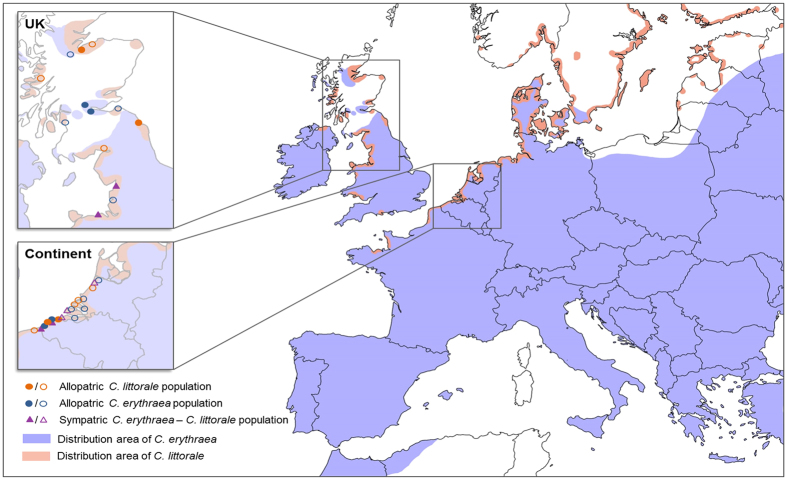
Map depicting the distribution of *Centaurium erythraea* and *C. littorale* and the location of the sampled populations in the UK and on the European mainland. Circles represent allopatric populations, whereas triangles denote sympatric populations. Full symbols denote populations where pollination experiments and floral measurements were conducted, whereas open symbols denote populations where only floral traits were measured. Map modified with Adobe Photoshop CS3 from OpenStreetMap.org. This OpenStreetMap is made available under the Open Database License: http://opendatacommons.org/licenses/odbl/1.0/. Any rights in individual contents of the database are licensed under the Database Contents License: http://opendatacommons.org/licenses/dbcl/1.0/.

*Centaurium erythraea* and *C. littorale* are biennial, monocarpic herbs belonging to the Gentianaceae. *Centaurium littorale* is often found in young, wet dune slacks, prefers moist and saline conditions and is a flood resistant species^37^,^38^. Its distribution is restricted to coastal areas from southern Scandinavia to the UK, France and Eastern Europe(Fig. 1). In contrast, *C. erythraea* is tolerant of a wider range of environmental conditions and can be found in a much broader variety of habitats, including coastal dunes, river banks, wood margins and calcareous grasslands. Distributed over most of Europe, its southern and eastern range ends in North Africa and Afghanistan, respectively^39^(Fig. 1). In coastal areas, the distribution of both species frequently overlaps, resulting in sympatric populations at many locations throughout the distribution range of *C. littorale*^34^,^37^,^38^,^39^(Fig. 1).

For both species, first year juveniles overwinter as a basal rosette and generally flower in the next growing season. Both species produce numerous flowers that show a comparable floral morphology on one or more flowering stalks. *C. erythraea* produces showy, pink flowers while *C. littorale* flowers are often mauve. Once the flowers open, they last for 4 to 5 days^35^,^36^. Both species flower from the end of June until the beginning of September^33^. Flowers of both species do not produce any nectar and largely share the same pollinator community. Insects reported to visit the flowers of both species include pollen-gathering hoverflies(Diptera, Syrphidae), bees(Hymenoptera, Apidae) and small flies(Empididae-Muscidae)^11^,^35^. If a flower is successfully pollinated, fruits produce large amounts(241.1 ± 51.7 and 206.5 ± 42.8 seeds per fruit in *C. erythraea* and *C. littorale* respectively) of tiny seeds(<0.01 mg)^35^. When successful pollinator-mediated pollination fails, both species are able to set fruit through autonomous self-pollination. *C. erythraea* and *C. littorale* are self-compatible and spontaneous selfing occurs through anther-curling^35^. Because of incomplete reproductive isolation, both species have been shown to hybridise in the field^33^,^34^,^36^.

### Study area and populations

To assess geographical variation in flower morphology and to investigate the impact of interspecific interference on floral traits, 36 populations of *C. erythraea* and *C. littorale* were studied across two disjunct geographic regions in Europe. Twenty-one populations were located on mainland Europe(along the coast of Belgium and the Netherlands), whereas fifteen populations were sampled in the UK(Scotland, northern Wales and England)(Fig. 1, Table 1). Continental populations included six allopatric *C. erythraea,* five allopatric *C. littorale* populations, and ten sympatric populations. In the UK, six allopatric *C. erythraea*, five allopatric *C. littorale* populations, and four sympatric populations were sampled(Table 1). All populations were sampled in coastal areas and dune slacks of the two regions, so that all locations were similar, i.e. dominated by saline and moist conditions. All allopatric populations were distinct entities that occurred at least 500 meters from another population of the same or the other species, whereas in sympatric populations, plants of both species grew intermixed. The populations studied in this work counted on average 500 plants, with maximum and minimum population sizes around 2000 and 200, respectively.

### Floral measurements

**Figure 2:**
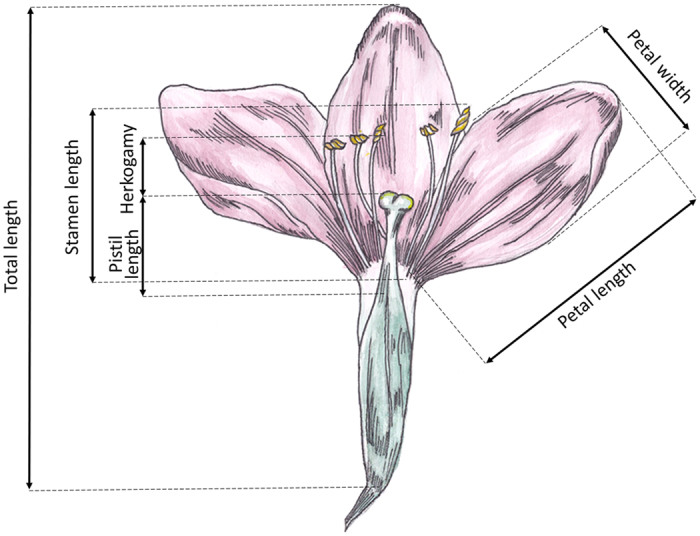
Lateral view of a sectioned *Centaurium* flower. All floral measurements that were made to characterize variation in floral morphology of flowers sampled in allopatric and sympatric populations of *Centaurium erythraea* and *C. littorale* are indicated.

In each population, variation in floral traits was stable, so we selected 30 plants and of each individual one freshly opened flower was collected during peak flowering. Each of these flowers were opened longitudinally and scanned to obtain a cross-section of the flower that was used for floral measurements. For each flower, six floral traits(total flower length, petal length, petal width, pistil length, stamen length and the degree of herkogamy) were measured using ImageJ software^40^(Fig. 2). Herkogamy was determined as the distance between the pistil and the first anther. Because previous research^35^ has shown that these floral traits do not vary within the same plant, harvesting a single flower per plant was sufficient to characterize variation in overall floral morphology among populations and species.

### The contribution of autonomous selfing to total seed production under natural field conditions

To quantify the extent of pollinator-mediated seed set and to assess the contribution of autonomous selfing to total seed production, floral manipulations were performed using methods outlined in Eckert *et al*.^7^ in two allopatric and two sympatric populations per species in each geographic region(UK and the European mainland)(12 sites, 16 populations in total). In each population, we selected and marked 30 similar sized plants throughout the population representing the average size and floral display of that particular population. For each individual, plant height, the number of flowers and the number of flowering stalks was measured. Afterwards, two pollination treatments were applied on eight flowers in total. Four flowers were emasculated immediately after anthesis and before anthers dehisced to obtain an estimate of pollinator-mediated seed production, whereas the other four flowers were left unmanipulated and served as controls. Each of these flowers were exposed to open natural pollination in the field. Because only a few flowers per plant were assigned to the emasculation treatment in this study, we assumed that emasculation did not impact plant attractiveness and pollinator services in this experiment. Moreover, previous studies have indicated that emasculation of flowers in both species did not affect the average life span of a flower nor floral visitation rates^35^,^41^. These studies also suggest that pollinator behavior on flowers is unlikely to be affected by emasculation, so that all floral visits are of similar quality.

Once fruits were ripe, each marked fruit was harvested and the number of seeds per fruit was counted. For each plant, average fruit-level seed production per treatment was determined by calculating the average number of seeds. With these data, we also calculated for each plant an index of reproductive assurance(*RA*) as [1 –(seed set of emasculated flowers/seed set of intact flowers)]^7^,^42^.

### Data analysis

To test whether overall floral morphology differed between allopatric and sympatric populations and whether these differences were affected by region and species, a multivariate analyses of variance(MANOVA) was applied using the raw data of floral measurements, with region, species and allopatric/sympatric populations and their interactions as fixed effects. The three-way interaction was also included in the model. Subsequently, two-way MANOVAs were conducted for each region separately to investigate in more detail how co-occurrence of the two species affected their overall morphology. In this analysis, species and whether populations were allopatric or sympatric as well as their interaction were used as explanatory variables in the model. Subsequently, univariate analyses of variance were performed per species to see which of the six floral traits differed significantly between population type.

To investigate whether differences in floral traits between the two species were larger in sympatric than in allopatric populations, we calculated for each floral trait the average difference between population means of the two species. The average difference was calculated for all combinations of *C. erythraea* and *C. littorale* populations, and done separately for allopatric and sympatric populations. These averages display the magnitude of the difference between the two species in allopatry and sympatry. Finally, the average differences in floral traits between both species were compared between allopatric and sympatric populations. All calculations were done separately for populations on the European mainland and the UK. To compare the average difference in plant height, number of flowers and flowering stalks with differences in floral traits and reproductive assurance, we used the same calculations for average difference as in floral traits, mentioned above. All measurements are represented as means and standard errors.

To test whether the contribution of autonomous selfing to total seed production(i.e., RA) was correlated with any floral traits, we calculated the Pearson correlation coefficient for all combinations of RA and the six floral traits. To test whether the contribution of autonomous selfing to total seed production(i.e., RA) differed between allopatric and sympatric populations and whether these differences were affected by region and species, we used a generalized linear mixed model(GLMM) with the index of reproductive assurance(RA) as dependent variable and region, species, population type(allopatry/sympatry) as fixed effects. Interaction effects were also included in the model and population was included as a random factor. This analysis was conducted in SAS 9.1(SAS Institute, 2005), with the GLIMMIX procedure and logit link function. All other analyses were performed using SPSS^43^ and R^44^. All graphs were made with Sigmaplot(Systat Software, San Jose, CA).

## Results

### Floral morphology

The results of the MANOVA showed significant(*P* < 0.05) differences in floral morphology between *C. erythraea* and *C. littorale*, between allopatric and sympatric populations and between regions(Table 2). Moreover, interactions between these factors were significant as well, indicating that differences in floral morphology were largely context-dependent. Region had by far the largest effect on overall morphology(Table 2), followed by species and their interaction, indicating that the two species differed significantly in floral morphology depending on the region where they were growing. Interestingly, whether a species was growing in allopatric or sympatric populations also significantly affected floral morphology, and this effect in turn interacted with region and species. Finally, the three-way interaction between region, species and allopatric/sympatric populations was also significant(Table 2).

**Figure 3:**
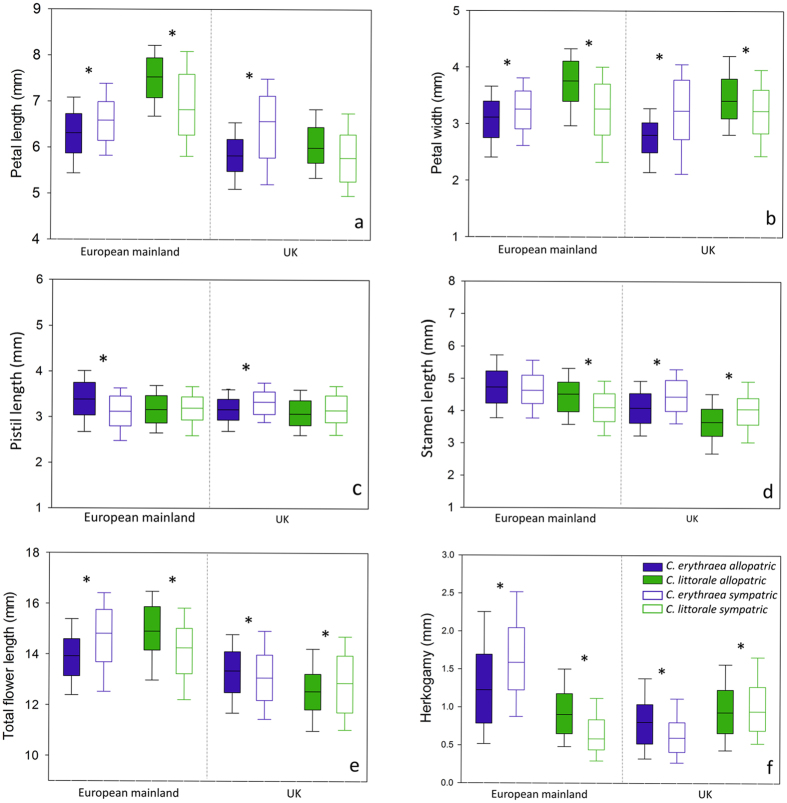
Morphological characteristics between allopatric and sympatric populations of *Centaurium erythraea* and *C. littorale* from the European mainland and the UK. An asterisk indicates significant differences between the characteristics in allopatry and sympatry.

Analyses for each region separately showed that floral morphology differed significantly between plants of allopatric and sympatric populations, and that this effect was dependent on species(Table S1). Univariate analyses of variance showed that total length(*F*_1,268_ = 12.41; *P* = 0.001), petal length(*F*_1,268_ = 59.74; *P* < 0.001) and petal width(*F*_1,268_ = 69.54; *P* < 0.001) of *C. littorale* flowers on the European mainland were significantly smaller in sympatric than in allopatric populations, indicating that flowers of *C. littorale* in sympatric populations were in general smaller than those in allopatric populations(Fig. 3, Table S2). In contrast, total length(*F*_1,369_ = 37.76; *P* < 0.001), petal length(*F*_1,369_ = 51.37; *P* < 0.001) and petal width(*F*_1,369_ = 37.53; *P* < 0.001) were significantly larger in sympatric populations of *C. erythraea* than in allopatric conditions, indicating that flowers of *C. erythraea* are larger under sympatric conditions(Fig. 3, Table S2). The univariate analyses of variance further showed that in allopatric populations, *C. erythraea* flowers showed a higher degree of herkogamy than flowers of *C. littorale*, and this distinction significantly increased in sympatric populations with a significant increase of herkogamy in *C. erythraea(F*_1,369_ = 79.20; *P* < 0.001) and a decrease in *C. littorale(F*_1,268_ = 21.69; *P* < 0.001)(Fig. 3, Table S2).

Results for populations in the UK showed that petal length(*F*_1,240_ = 40.17; *P* < 0.001) and width(*F*_1,240_ = 49.80; *P* < 0.001) of the flowers were significantly larger in sympatric than in allopatric populations for *C. erythraea* populations, while *C. littorale* flowers showed a significant decrease from allopatric to sympatric flowers for petal width(*F*_1,243_ = 44.25; *P* < 0.001)(Fig. 3, Table S2). Despite this, sympatric populations of *C. erythraea* were significantly smaller in total length(*F*_1,240_ = 37.13; *P* < 0.001) than allopatric populations and total length(*F*_1,243_ = 7.59; *P* = 0.006) of *C. littorale* increased from sympatric to allopatric populations. Pistil length did only differ between sympatric and allopatric populations of *C. erythraea,* with sympatric populations being significantly larger(*F*_1,240_ = 12.03; *P* = 0.001), whereas stamen length increased in sympatric populations of *C. erythraea(F*_1,240_ = 18.19; *P* < 0.001) and *C. littorale(F*_1,243_ = 5.26; *P* = 0.023)(Fig. 3, Table S2). The level of herkogamy, on the other hand, decreased significantly in sympatric populations of *C. erythraea(F*_1,240_ = 18.86; *P* < 0.001), and increased slightly in sympatric populations of *C. littorale(F*_1,243_ = 5.47; *P* = 0.020).

**Figure 4:**
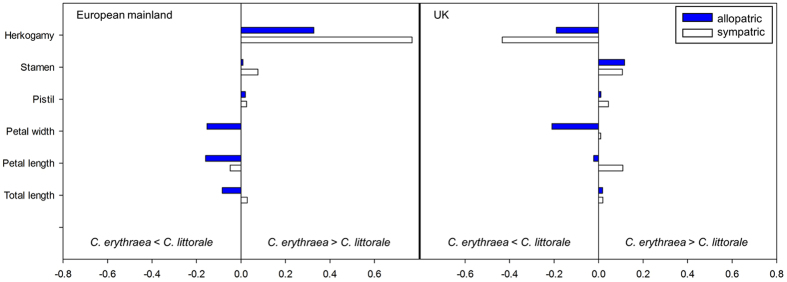
Relative differences of floral traits, calculated between *Centaurium erythraea* and *C. littorale* for allopatric and sympatric populations separately on the European mainland and the UK. Negative values indicate that the floral trait is larger in *C. littorale* while positive values show that floral traits in *C. erythraea* are the largest.

In both regions, the difference in herkogamy between the two species was substantially larger in sympatric populations than in allopatric populations(Table 3, Fig. 4). On the European mainland, the difference in herkogamy between the two species increased from 0.38 mm ± 0.08 mm in allopatric populations to 0.82 mm ± 0.06 mm in sympatric populations. The same effect was seen in the UK, where the difference in herkogamy between the species increased from 0.19 mm ± 0.04 mm in allopatry to 0.37 mm ± 0.01 mm in sympatry. This indicates that the co-occurrence of the two species magnifies the difference in herkogamy(Fig. 4). On the European mainland, this difference is primarily driven by differences in stamen length, which increased from 0.04 mm ± 0.12 mm in allopatry to 0.33 mm ± 0.08 mm in sympatry. In the UK, on the other hand, differences in stamen length were similar between allopatric and sympatric populations, but variation in pistil length was mainly responsible for the variation in herkogamy and increased from 0.03 mm ± 0.06 mm in allopatry to 0.14 mm ± 0.03 mm in sympatry(Fig. 4).

Plant height, number of flowers and flowering stalks did also differ between allopatric and sympatric conditions(Table S3). On the European mainland, the difference in plant height increased from 9.31 cm ± 2.15 cm in allopatric populations to 13.65 cm ± 3.55 cm in sympatric populations, indicating that the two species tend to be more different in height when they co-occur. The average difference in the number of flowers and flowering stalks decreased from allopatric to sympatric populations, indicating that the two species tended to be more similar for these characters under sympatric conditions. In the UK, differences in these three characters decreased from allopatric populations to sympatric populations, indicating that the plants were more similar in sympatry.

### The contribution of autonomous selfing to total seed production

**Figure 5:**
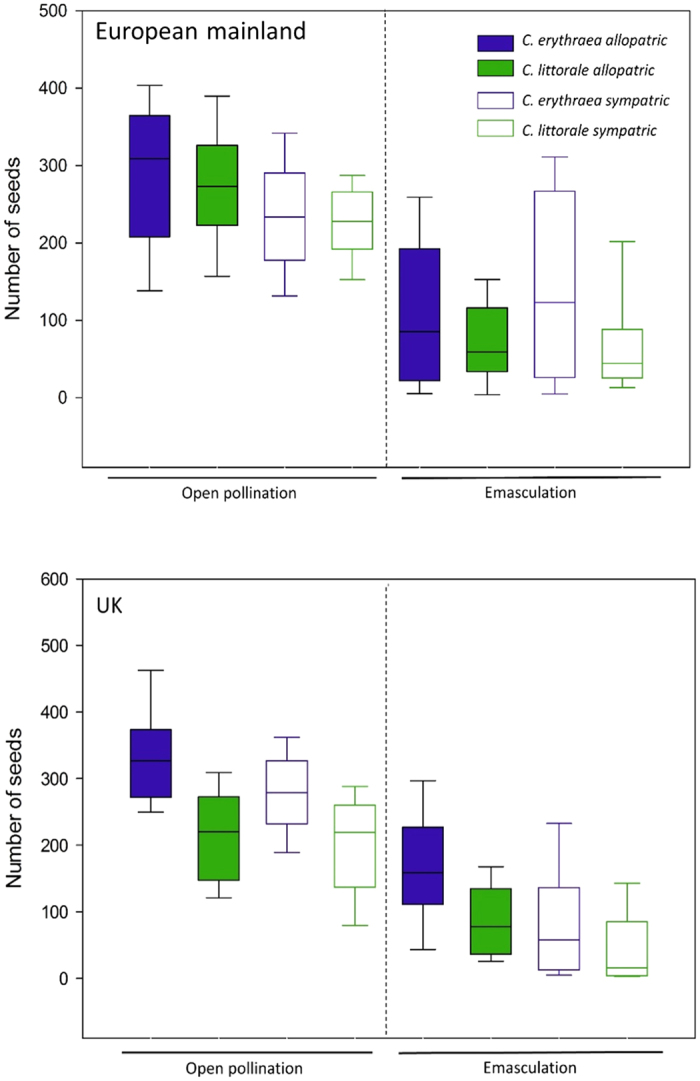
Seed set of emasculated and open pollinated flowers in sympatric and allopatric populations on the European mainland(top) and the UK(bottom).

Seed set differed significantly(*F*_1,902_ = 760.99; *P* < 0.001) between emasculated and open pollinated flowers(Fig. 5). Intact open pollinated flowers generally produced more seeds per fruit(on average 248.3 ± 4.1 seeds) compared to emasculated flowers that only produced on average 106.4(±4.7) seeds per fruit. This resulted in overall reproductive assurance value of RA = 0.65 ± 0.02, and this capacity to self autonomously was significantly related to the degree of herkogamy(*r* = −0.708; *P* = 0.049, *N* = 8), but not to the other floral traits. Furthermore, the extent to which autonomous selfing contributed to total seed production, depended significantly on species(*F*_*1,212*_ = 7.20; *P* = 0.008) and was in general higher for *C. littorale*(RA = 0.68 ± 0.03) than for *C. erythraea*(RA = 0.60 ± 0.03). The species specific contribution of autonomous selfing to total seed production also interacted significantly with geographic region(*F*_*1,212*_ = 14.77; *P* = 0.0002), with *C. erythraea* showing an increase in the contribution of autonomous selfing in the UK(RA = 0.67 ± 0.03) compared to the European mainland(RA = 0.48 ± 0.06), whereas the opposite pattern that was found in *C. littorale*. The contribution of autonomous selfing in *C. littorale* increased from 0.64(±0.04) in the UK to 0.76(±0.03) on the European mainland. We also found a significant interaction between species and whether the population is allopatric or sympatric(*F*_1,212_ = 8.51; *P* = 0.004). The contribution of autonomous selfing to total seed production was lower in sympatric(RA = 0.56 ± 0.05) than in allopatric(RA = 0.66 ± 0.04) populations of *C. erythraea*, while *C. littorale* showed the opposite pattern and had a higher contribution of autonomous selfing in sympatric(RA = 0.74 ± 0.03) than in allopatric populations(RA = 0.62 ± 0.04).

## Discussion

### Geographic variation in floral morphology and the capacity of autonomous selfing

**Figure 6:**
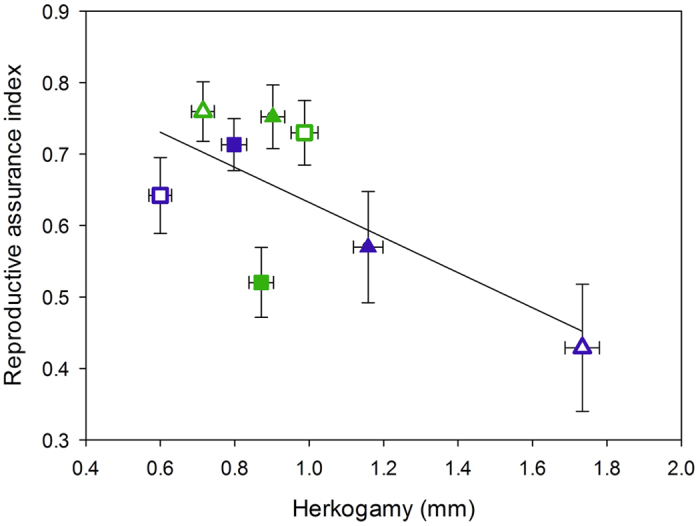
The relationship between herkogamy and the index of reproductive assurance(*RA*). Blue symbols represent *C. erythraea*, green ones *C. littorale.* Populations from the European mainland and the UK are represented by triangles and squares, respectively. Sympatric populations have hollow symbols and allopatric populations have filled symbols.

We found substantial geographic variation in floral traits in the two closely related *Centaurium* species studied here. Flowers of both species were on average larger on the European mainland than in the UK. The level of herkogamy was largest on the European mainland for *C. erythraea* whereas flowers of *C. littorale* showed higher levels of herkogamy in the UK. These results confirm earlier findings of Ubsdell^36^ and Brys and Jacquemyn^35^, who already showed that the flower morphology of these two *Centaurium* species differed between these two geographic regions. The observed shifts in the level of herkogamy between allopatric and sympatric populations also translated into different capacities to self spontaneously. Interestingly, autogamy in sympatry was achieved by different means in mainland vs island populations. In the UK, reduced herkogamy was realized by an increase in pistil length, whereas on the mainland higher autonomous selfing was obtained through a reduction in stamen length. These results therefore suggest that character displacement occurred independently on the mainland and island. Although the spatial separation of anthers and stigmas was quite small in both study species(ranging between 0.06 and 5.59 mm), it had a significant impact on a plants’ capacity to reproduce autonomously(Fig. 6), which varied between 0 to 99.7% seed set realized via autonomous selfing.

Herkogamy and flower size are plastic traits that can vary among sites, and some studies have shown that this can be correlated with drought stress^45^,^46^,^47^. In dryer regions, plants tend more to self-fertilization with faster flowering time, shorter period of anthesis, smaller flowers and lower herkogamy. Based on monthly weather data obtained from weather stations nearby the study populations, we can conclude that, apart from some minor differences in temperature, the two regions are characterized by similar climatic conditions, most likely as a result of their close proximity to the sea. Although we cannot completely exclude such an effect as explanatory factor, the studied populations were all taken from similar coastal habitats and even if a drought-related effect would take place, differences among allopatric and sympatric populations are still visible.

Differences in floral and mating system traits between the two regions may, on the other hand, also be driven by differences in pollinator communities. Work in a variety of systems has indeed shown that flowers tend to become smaller and evolve towards selfing at the edge of their distribution ranges due to pollen limitation, unstable populations and/or changing environments^2^,^29^,^48^,^49^. This trend was visible for *C. erythraea*, but less for *C. littorale*. It is therefore tempting to suggest that the observed differences in floral traits and capacity for autonomous selfing between the two species could be the result of differences in pollinator communities and abundances between the two regions. Given that the distribution of *C. littorale* is largely restricted to the coastal areas of Western Europe(Fig. 1), this species may have adapted its floral morphology to the specific pollinator communities that characterize coastal areas, and which are likely to be quite similar throughout most of its distribution area. *C. erythraea*, on the other hand, shows a much larger distribution area, which encompasses a large part of the Mediterranean and Continental Europe. It can therefore be assumed that this species is exposed to more diverse pollinator communities across its distribution area. *C. erythraea* populations in the UK also represent the marginal habitat of this species, while *C. littorale* can be found on even more northern coastlines in Europe(Fig. 1).

On the European mainland, a much larger number of insect species generally visits *C. erythraea* than *C. littorale*, and the number of visits per insect species is significantly higher in *C. erythraea* than in *C. littorale*^41^. Assuming that the abundance of some of these insects declines with increasing latitude, differences in pollinator communities between continental and British populations or differences in the abundance of specific pollinators may have triggered the evolution towards selfing in northern populations of *C. erythraea*. Given the large geographic barrier between the European mainland and the British Isles, differences in selection pressures on floral traits between both regions are most likely not counterbalanced by gene flow, leading to significant genetic and morphological divergence between the two regions. However, to unambiguously show that differences in pollinator communities underlie the observed differences in floral morphology and mating systems between regions, more detailed research on the pollinator communities is needed. This includes comparative pollinator surveys over the geographic gradient to test for differences and field experiments where plants are introduced from a different region to look into the effect of the local and foreign pollinator community.

### Floral divergence between allopatric and sympatric populations

In addition to pollinator-mediated floral divergence, differences in floral traits may result from inter-specific competition for pollinator services(i.e., reproductive character displacement) and/or selection against heterospecific mating and low fitness of the resulting hybrids(i.e., reinforcement)^19^,^23^,^29^,^33^. Under these conditions, it can be predicted that sympatric populations should display greater divergence in reproductive traits than their congeners in allopatric populations^21^. In some cases, the evolution to selfing will be favoured when a species co-occurs with a more outcrossing sister species, as the reduced herkogamy and an associated increase in the capacity to self autonomously will safeguard the species from heterospecific pollen transfer and hybrid formation. Previous work in a sympatric population of both *Centaurium* species in Belgium has shown that when the more selfing *C. littorale* is flowering in close proximity with the more outcrossing *C. erythraea*, the former is exposed to a significantly higher risk of heterospecific mating^33^. Moreover, the same research has shown that hybridization in *Centaurium* was costly^33^. The observed differences in floral traits between allopatric and sympatric populations may therefore be the result of selection towards higher autonomous selfing in order to reduce interspecific gene flow.

### A geographic mosaic of floral traits and mating systems

In case of the studied *Centaurium* species, we hypothesize that when *C. erythraea* colonized the British Isles from the Continent after the last Ice Age, a shift to a more selfing mating system may have occurred in order to ensure sufficient reproductive assurance. When it was exposed to *C. littorale*, mating systems evolved further to selfing to reduce interspecific mating. On the European mainland, on the other hand, *C. erythraea* is characterized by a predominantly outcrossing mating system. When coming into contact with more selfing *C. littorale*, divergence in mating systems became reinforced so that *C. littorale* became more selfing in sympatric populations. Given the much more restricted distribution of *C. littorale* and its close affinity to coastal habitats(Fig. 1), it is reasonable to assume that *C. littorale* did not shift to higher selfing when it re-colonised the UK.

## Conclusion

In this study, we confirmed earlier findings that the two *Centaurium* species displayed substantial variation in floral morphology across different geographic regions. This variation was also reflected in the contribution of autonomous selfing to total seed production, which largely paralleled the geographical variation in floral traits. Overall, these results reinforce the idea that floral traits related to mating systems can vary substantially depending on the ecological context in which the population occurs. Moreover, our study demonstrates that the joint occurrence of closely related plant species can have an important impact on floral morphology and the capacity of autonomous selfing, and suggests that reinforcement may be an important factor driving the evolution and maintenance of mating systems in sympatric populations. However, to fully understand the drivers of mating system variation in closely related plant species and to demonstrate that the evolution towards increased selfing reduces hybridization, reciprocal transplant experiments between allopatric and sympatric populations and across the studied geographic regions should be performed in the future and the fitness of their progeny assessed.

## Additional Information

**How to cite this article**: Schouppe, D. *et al*. Geographic variation in floral traits and the capacity of autonomous selfing across allopatric and sympatric populations of two closely related *Centaurium* species. *Sci. Rep.* **7**, 46410; doi: 10.1038/srep46410(2017).

**Publisher's note:** Springer Nature remains neutral with regard to jurisdictional claims in published maps and institutional affiliations.

## Supplementary Material

Supplementary Information

## Figures and Tables

**Table 1:** Morphological characteristics(mean ± SE), sample size and geographic coordinates of allopatric and sympatric populations of *Centaurium erythraea* and *C. littorale* sampled in the UK and on the European mainland.

Species	Population type	Name	^#^samples	Eastern longitude	Northern latitude	Total length(mm)	Petal length(mm)	Petal width(mm)	Pistil length(mm)	Stamen length(mm)	Herkogamy(mm)
**European mainland**											
*C. erythraea*	allopatric	De Haan	88	3° 4′1″	51°17′32″	13.41 ± 0.11	5.97 ± 0.06	2.95 ± 0.06	3.74 ± 0.04	5.17 ± 0.07	1.18 ± 0.06
		De Klip	19	4° 22′ 7″	52° 8′ 38″	14.12 ± 0.11	6.59 ± 0.06	3.33 ± 0.06	3.45 ± 0.08	4.72 ± 0.11	1.86 ± 0.07
		Grenspad	76	2° 33′ 12″	51° 4′ 48″	14.62 ± 0.19	6.42 ± 0.09	2.97 ± 0.05	3.05 ± 0.05	4.63 ± 0.08	1.54 ± 0.10
		Jacobahaven	20	3° 41′ 23″	51° 36′ 1″	14.28 ± 0.08	6.41 ± 0.06	3.08 ± 0.05	3.47 ± 0.04	4.50 ± 0.11	1.59 ± 0.09
		Oostdijk	20	3° 58′ 7″	51° 49′ 47″	13.92 ± 0.21	6.68 ± 0.11	3.63 ± 0.08	2.99 ± 0.06	4.07 ± 0.12	0.87 ± 0.10
		Terneuzen	20	3° 44′ 27″	51° 20′ 5″	13.40 ± 0.21	6.55 ± 0.12	3.07 ± 0.07	3.17 ± 0.13	4.24 ± 0.12	0.85 ± 0.07
	sympatric	Groenplein	16	3° 19′ 10″	51° 21′ 22″	14.67 ± 0.37	7.06 ± 0.18	3.07 ± 0.16	3.52 ± 0.05	4.39 ± 0.12	1.15 ± 0.09
		Meijendel	20	4° 21′ 4″	52° 8′ 14″	14.04 ± 0.18	6.24 ± 0.08	3.18 ± 0.08	3.37 ± 0.06	4.09 ± 0.09	1.24 ± 0.11
		Schotsman	20	3° 41′ 17″	51° 34′ 23″	14.81 ± 0.19	6.66 ± 0.12	3.56 ± 0.07	3.46 ± 0.08	4.30 ± 0.14	1.52 ± 0.09
		Ter Yde	82	2° 41′ 53″	51° 8′ 10″	14.81 ± 0.16	6.56 ± 0.07	3.15 ± 0.06	3.01 ± 0.05	4.60 ± 0.07	1.64 ± 0.06
		Westhoek	143	2°34′3″	51° 5′29″	14.50 ± 0.16	6.55 ± 0.05	3.28 ± 0.04	3.02 ± 0.04	4.88 ± 0.06	1.83 ± 0.06
*C. littorale*	allopatric	Brouwersdam	20	3° 52′ 13″	51° 46′ 11″	15.95 ± 0.38	7.84 ± 0.13	3.82 ± 0.08	3.50 ± 0.07	4.81 ± 0.11	0.73 ± 0.07
		De Haan	79	3° 4′13″	51°17′45″	14.20 ± 0.13	7.42 ± 0.07	3.84 ± 0.06	3.07 ± 0.05	4.51 ± 0.06	0.99 ± 0.04
		De Punt	20	3°52′59″	51°46′48″	15.07 ± 0.24	7.27 ± 0.12	3.89 ± 0.07	3.51 ± 0.05	5.14 ± 0.09	1.09 ± 0.10
		Frankrijk	20	2° 31′ 45″	51° 4′ 56″	15.51 ± 0.30	7.71 ± 0.13	3.32 ± 0.09	3.06 ± 0.07	3.83 ± 0.12	0.95 ± 0.09
		Zwin	71	3° 19′ 59″	51° 21′ 41″	15.18 ± 0.14	7.47 ± 0.08	3.59 ± 0.06	3.10 ± 0.04	4.28 ± 0.08	0.94 ± 0.05
	sympatric	Groenplein	16	3° 19′ 10″	51° 21′ 22″	12.23 ± 0.44	5.76 ± 0.32	2.19 ± 0.17	2.86 ± 0.19	3.67 ± 0.19	0.79 ± 0.12
		Meijendel	20	4° 21′ 4″	52° 8′ 14″	15.20 ± 0.18	7.57 ± 0.12	4.11 ± 0.06	3.36 ± 0.04	4.31 ± 0.10	0.62 ± 0.07
		Schotsman	20	3° 41′ 17″	51° 34′ 23″	15.42 ± 0.25	7.90 ± 0.09	3.68 ± 0.06	3.55 ± 0.06	4.56 ± 0.13	0.44 ± 0.05
		Ter Yde	77	2° 41′ 53″	51° 8′ 10″	13.97 ± 0.14	6.63 ± 0.09	3.15 ± 0.05	3.10 ± 0.04	4.01 ± 0.07	0.67 ± 0.03
		Westhoek	61	2°34′3″	51° 5′29″	14.01 ± 0.16	6.88 ± 0.09	3.11 ± 0.07	3.11 ± 0.06	4.08 ± 0.07	0.76 ± 0.06
**UK**											
*C. erythraea*	allopatric	Bo′ness	60	−4° 22′ 27″	56° 0′ 57″	13.57 ± 0.12	5.61 ± 0.06	2.95 ± 0.04	3.30 ± 0.03	4.22 ± 0.07	0.75 ± 0.05
		Dirleton	19	−3° 11′ 45″	56° 3′ 27″	11.88 ± 0.23	5.65 ± 0.12	2.12 ± 0.09	2.63 ± 0.09	3.57 ± 0.14	0.73 ± 0.09
		Formby	19	−4° 54′ 12″	53° 33′ 58″	13.47 ± 0.29	5.99 ± 0.11	2.85 ± 0.09	3.15 ± 0.08	4.15 ± 0.14	1.18 ± 0.10
		Foyers	20	−5° 30′ 15″	57° 15′ 21″	12.58 ± 0.24	5.78 ± 0.10	2.81 ± 0.08	3.13 ± 0.07	3.99 ± 0.14	0.89 ± 0.05
		Irvine	20	−5° 20′ 36″	55° 35′ 54″	11.83 ± 0.21	5.78 ± 0.09	2.92 ± 0.15	3.11 ± 0.06	3.65 ± 0.15	0.86 ± 0.10
		Roslin	80	−4° 49′ 17″	55° 51′ 32″	13.87 ± 0.11	6.05 ± 0.07	2.70 ± 0.05	3.17 ± 0.04	4.21 ± 0.08	0.79 ± 0.04
	sympatric	Ainsdale	80	−4° 56′ 42″	53° 37′ 7″	13.00 ± 0.14	5.72 ± 0.07	2.72 ± 0.06	3.19 ± 0.05	4.34 ± 0.06	0.62 ± 0.04
		Talacre	80	−4° 40′ 13″	53° 21′ 14″	13.18 ± 0.16	7.11 ± 0.07	3.67 ± 0.06	3.40 ± 0.04	4.58 ± 0.08	0.70 ± 0.04
*C. littorale*	allopatric	Holy island	80	−2° 10′ 5″	55° 41′ 4″	12.23 ± 0.13	5.82 ± 0.06	3.64 ± 0.06	3.20 ± 0.04	3.88 ± 0.06	0.79 ± 0.04
		Kentra	19	−6° 9′ 3″	56° 45′ 28″	11.51 ± 0.24	5.60 ± 0.12	2.98 ± 0.09	3.22 ± 0.07	2.90 ± 0.12	1.09 ± 0.09
		Kinloss	21	−4° 24′ 50″	57° 38′ 48″	12.93 ± 0.28	5.17 ± 0.10	3.26 ± 0.14	3.06 ± 0.06	3.45 ± 0.11	1.22 ± 0.12
		Nairn	80	−4° 10′ 4″	57° 35′ 46″	12.51 ± 0.11	6.14 ± 0.06	3.55 ± 0.06	3.03 ± 0.04	3.61 ± 0.08	1.06 ± 0.04
		Powfoot	19	−4° 38′ 5″	54° 58′ 17″	14.02 ± 0.25	6.97 ± 0.11	3.28 ± 0.08	2.74 ± 0.05	3.82 ± 0.12	1.12 ± 0.14
	sympatric	Ainsdale	68	−4° 56′ 42″	53° 37′ 7″	13.05 ± 0.14	5.53 ± 0.06	2.88 ± 0.08	3.15 ± 0.06	4.15 ± 0.08	1.00 ± 0.05
		Talacre	85	−4° 40′ 13″	53° 21′ 14″	12.64 ± 0.18	5.98 ± 0.08	3.45 ± 0.06	3.16 ± 0.05	3.87 ± 0.06	1.05 ± 0.05

**Table 2:** Results of the multivariate analysis of variance(MANOVA) using seven morphological traits as dependent variables and region(UK vs. European mainland), species(*Centaurium erythraea* and *C. littorale*) and population type(allopatric/sympatric population) and their interactions as independent factors.

Effect	Wilks′ λ	*F*	*P*	*df*
Intercept	0.007	32199.662	<0.0001	6
Region	0.652	127.066	<0.0001	6
Species	0.699	102.526	<0.0001	6
Population type	0.992	2.232	0.038	6
Region *Species	0.696	104.059	<0.0001	6
Region * Population type	0.910	23.621	<0.0001	6
Species *Population type	0.866	36.945	<0.0001	6
Region *Species *Population type	0.922	20.097	<0.0001	6

**Table 3:** Magnitude of differences and standard errors in morphological characters between allopatric and sympatric populations of *C. erythraea* and *C. littorale* sampled on the European mainland and the UK.

		Total length	Petal length	Petal width	Pistil length	Stamen length	Herkogamy
European mainland	allopatric	−1.22 ± 0.15	−1.11 ± 0.06	−0.52 ± 0.07	0.06 ± 0.07	0.04 ± 0.12	0.38 ± 0.08
	sympatric	0.4 ± 0.24	−0.33 ± 0.16	0 ± 0.14	0.08 ± 0.07	0.33 ± 0.08	0.82 ± 0.06
UK	allopatric	0.23 ± 0.24	−0.13 ± 0.13	−0.62 ± 0.08	0.03 ± 0.06	0.43 ± 0.09	−0.19 ± 0.04
	sympatric	0.24 ± 0.05	0.66 ± 0.17	0.03 ± 0.13	0.14 ± 0.03	0.45 ± 0.04	−0.37 ± 0.01
