# Supplementary material for: Geographic variation in floral traits and the capacity of autonomous selfing across allopatric and sympatric populations of two closely related Centaurium species
Source: Sci Rep. 2017 Apr 21;7:46410. doi: 10.1038/srep46410 (PMC5399455; doi:10.1038/srep46410)
Supplement: Supplementary Information [file srep46410-s1.pdf]

Geographic variation in floral traits and the capacity of autonomous selfing across allopatric and sympatric populations of two closely related *Centaurium* species

Dorien Schouppe[1*], Rein Brys[2], Mario Vallejo-Marin[3], Hans Jacquemyn[1]

[1]*KU Leuven, Department of Biology, Plant Conservation and Population Biology, B-3001 Leuven, Belgium*

[2]*Research Institute for Nature and Forest, Kliniekstraat 25, BE-1070 Brussels, Belgium*

[3]*Biological and Environmental Sciences. University of Stirling. Stirling, FK9 4LA. Scotland, United Kingdom.*

Correspondence and request for materials should be addressed to S.D. (dorien.schouppe@kuleuven.be)

Table S1 Multivariate analysis of variance (MANOVA), performed on populations of the UK and European mainland separately, on all floral traits with species and population type (allopatric/sympatric populations) and their interactions as fixed effects.

	UK			European mainland		
Effect	Wilks' λ	*F*	*P*	Wilks' λ	*F*	*P*
Intercept	0.007	18148.338	<0.0001	0.007	20855.364	<0.0001
Species	0.632	72.404	<0.0001	0.552	124.307	<0.0001
Population type	0.901	13.668	<0.0001	0.946	8.700	<0.0001
Species * Population type	0.822	26.923	<0.0001	0.819	33.854	<0.0001

Table S2 Univariate analyses of variance per region and species, performed on all floral traits with population type as fixed effect.

	European mainland						UK					
	C. erythraea			*C. littorale*			*C. erythraea*			*C. littorale*		
Effect	*F*	*P*	*df*	*F*	*P*	*df*	*F*	*P*	*df*	*F*	*P*	*df*
Total length	37.762	<0.0001	1	12.413	0.001	1	37.131	<0.0001	1	7.592	0.006	1
Petal length	51.365	<0.0001	1	59.734	<0.0001	1	40.172	<0.0001	1	1.815	0.179	1
Petal width	37.530	<0.0001	1	69.539	<0.0001	1	49.797	<0.0001	1	44.254	<0.0001	1
Pistil length	96.037	<0.0001	1	0.064	0.801	1	12.028	0.001	1	0.860	0.355	1
Stamen length	3.383	0.067	1	25.857	<0.0001	1	18.189	<0.0001	1	5.264	0.023	1
Herkogamy	79.203	<0.0001	1	21.687	<0.0001	1	18.856	<0.0001	1	5.465	0.020	1

Table S3 Magnitude of differences and standard errors in plant height, number of flowers and flowering stalks between allopatric and sympatric populations of *C. erythraea* and *C. littorale* sampled on the European mainland and the UK.

		Plant height	Number of flowers	Number of flowering stalks
European mainland	allopatric	9.31 ± 2.15	60.62 ± 21.31	-7.03 ± 1.48
	sympatric	13.65 ± 3.55	36.15 ± 3.17	-1.90 ± 0.57
UK	allopatric	20.50 ± 2.17	88.83 ± 14.66	0.93 ± 0.40
	sympatric	11.75 ± 1.99	35.10 ± 11.15	0.17 ± 0.22
